# Emergency Medical Service Responses for Older Adults: A Retrospective Observational Study Comparing Nursing Homes and the Community

**DOI:** 10.3390/healthcare13212806

**Published:** 2025-11-05

**Authors:** Christine Gaik, Hinnerk Wulf, Valesco Mann, Dennis Humburg, Benjamin Vojnar

**Affiliations:** 1 Faculty of Medicine, Department of Anesthesiology and Intensive Care Medicine, Marburg University, Baldingerstrasse, 35033 Marburg, Germany; 2 Department of Anesthesiology and Intensive Care Medicine, University Hospital Giessen and Marburg, Campus Marburg, Baldingerstraße, 35043 Marburg, Germany; 3 Department of Anaesthesiology, Intensive Care Medicine, and Pain Therapy, Agaplesion Evangelical Hospital Mittelhessen, 35398 Giessen, Germany; 4 Department for Emergency Medicine, University Hospital Giessen and Marburg, Campus Marburg, 35043 Marburg, Germany; 5 Department of Hazard Prevention and Emergency Service, District of Vogelsberg, Goldhelg 20, 36341 Lauterbach, Germany

**Keywords:** Emergency Medical Services (EMS), geriatric patients, nursing homes, older patients, prehospital care

## Abstract

**Background**: Older adults (≥65 years) account for a substantial share of Emergency Medical Service (EMS) activations, yet differences between nursing homes (NHs) and the community are insufficiently defined. This study aimed to compare EMS responses for older adults inside and outside NHs. **Methods**: We conducted a retrospective observational study of all EMS activations involving patients ≥65 years in a rural German region from July 2020 to March 2025, based on fully anonymized prehospital patient care reports electronically documented on tablets. Prehospital patient care was assessed using the ABCDE approach, with additional analysis of feedback codes transmitted to the control center (response and on-scene times, physician dispatch, lights and sirens use, feedback codes, hospital destination, and admission urgency). Continuous variables were summarized as mean (SD) or median [IQR], categorical variables as *n* (%), and group comparisons performed using the Chi-Square test or Fisher’s exact test (categorical) and the t test or Mann–Whitney U test (continuous), as appropriate. **Results**: Among 46,598 EMS activations in adults aged ≥65 years, 28,749 EMS responses were included in the analysis after excluding non-emergency transports and duplicate physician records. Of these, 20% occurred in NHs (5707/28,749) and 80% in the community (23,042/28,749). Median age was 85 years (IQR 80–89) in NH patients and 80 years (IQR 73–86) in community patients (*p* < 0.001). Females accounted for 60% (3450/5705) of NH patients and 53% (12,223/23,036) of community patients (*p* < 0.001). Emergency physicians were dispatched in 7% of NH incidents (392/5707) and 10% of community incidents (2327/23,042; *p* < 0.001). In NHs, bronchitis/pneumonia was a leading feedback code (6%, 354/5707), whereas in community patients, acute coronary syndrome (ACS) was prominent (5%, 1249/23,042). Admission urgency categories also differed significantly, with NH residents classified as category 3 (outpatient evaluation) in 11% (621/5706) and category 0 (no admission) in 5% (280/5706), whereas community patients were more often classified as category 1 (immediate intervention) in 13% (2886/23,037) (all *p* < 0.001). **Conclusions**: NH residents were older, more often female, and presented with low-to-moderate acuity. Frequent diagnoses were closed extremity injuries and bronchitis/pneumonia. In contrast, ACS and other cardiovascular emergencies were more common in the community, particularly among men, consistent with international evidence on sex-specific incidence. NH residents were more often classified as non-urgent or outpatient and transported to nearby hospitals, whereas community patients more frequently required immediate intervention and referral to tertiary centers. In summary, EMS responses for older adults differed in clinical presentations, operational patterns, and hospital pathways.

## 1. Introduction

The growing proportion of older adults in Germany is a defining aspect of the country’s demographic change. According to official population statistics, the number of individuals aged 65 years and older increased from 12 million in 1991 to 18.7 million in 2022, corresponding to a rise from 15% to 22% of the total population [1]. In some rural areas, the proportion of residents aged ≥65 years exceeds 30% [2], highlighting substantial regional differences in population aging.

Living arrangements in later life vary considerably. Approximately 96% of people aged ≥65 years in Germany live in private households, whereas only about 4% reside in nursing homes (NHs), residential care facilities, or similar communal living arrangements [3]. However, the likelihood of living in a NH increases markedly with age: among people in need of care, nearly 16% of those aged 65–69 years live in NHs, compared with 26% of those aged ≥80 years and 35% of those aged ≥90 years [3]. These patterns illustrate not only the diversity of living situations but also the potential differences in healthcare needs between older adults in NHs and those living elsewhere.

In Germany, out-of-hours medical services are available to provide urgent primary care, but their use remains limited, even though they could help to buffer avoidable hospital admissions in NH residents [4]. As a result, EMS is often the first point of contact when acute health issues arise in this population. Understanding how EMS encounters in NHs compared with those in the community, and how they intersect with alternative care pathways, is therefore essential.

Older adults are known to account for a substantial proportion of EMS activations; however, relatively few studies have examined EMS utilization specifically in patients aged ≥65 years. Evidence indicates that this group uses EMS more frequently than younger individuals, with NH residents representing a subgroup with particularly high prehospital care needs [5]. In one of our previous studies, most NH-related EMS responses involved patients with stable vital signs and non-urgent conditions, with falls—especially suspected hip fractures—being the leading reason for EMS activation [6].

Against this background, we conducted a retrospective analysis of EMS activations in Vogelsbergkreis, a rural district in Germany, where the proportion of residents aged ≥65 years is 26% and above the state average [7]. We therefore aimed to compare EMS responses inside versus outside NHs in patients aged ≥65 years over a period of 4 years and 8 months, with respect to response and on-scene times, physician dispatch, use of lights and sirens, assessment using the ABCDE approach, feedback-code patterns, hospital destination, and admission urgency categories.

## 2. Materials and Methods

### 2.1. Study Design and Ethics

This retrospective observational study analyzed all EMS responses involving patients aged ≥65 years in Vogelsbergkreis (Hesse, Germany) between July 2020 and March 2025. In accordance with German regulations, no prior ethics approval was required to initiate data collection, as all EMS documentation was part of routine clinical practice and fully anonymized. However, formal authorization to access and scientifically analyze the existing anonymized dataset was subsequently granted by the Ethics Committee of the Medical Faculty, Philipps University of Marburg (Ref. No. 25-229-RS, approval date: 12 August 2025, chaired by Prof. Dr. Carola Seifart) and registered in the German Clinical Trials Register (DRKS-ID DRKS00037536). The study was conducted in accordance with the Declaration of Helsinki. As anonymized routine data were used, individual consent was not required. Reporting follows the Strengthening the Reporting of Observational Studies in Epidemiology (STROBE) guidelines.

### 2.2. Setting

#### 2.2.1. Region

Vogelsbergkreis is a rural district in central Germany, located north of Frankfurt. It covers an area of 1460 km^2^ (563 square miles) with 19 municipalities (7 towns) and a population of approximately 106,792 residents [8,9]. Population density is 73/km^2^ (vs. 273/km^2^ nationally) [10]. In 2023, the average age in Vogelsbergkreis was 47 years, the median age was 50 years, and about 26% of residents were aged ≥65 years, which is above the national average [11]. On a national level, Germany’s median age reached 45 years in 2024, with 22% of residents aged ≥65 years [12]. Across the European Union, the median age was 45 years in 2023, and 22% of the population was aged ≥65 years [13].

#### 2.2.2. Public Health

The district includes 24 NHs, about 72 general practitioners across approximately 48 practice locations, alongside four hospitals; EMS coverage comprises 14 ambulances and three physician response vehicles stationed at 12 bases.

#### 2.2.3. EMS System

Previous studies demonstrated substantial heterogeneity in prehospital EMS across Europe [14]. In some countries, physicians play a key role in emergency care, whereas in others, this role is carried out by specialized non-physician professionals, such as paramedics [15]. In Germany, emergency calls are managed by a central dispatch center, which decides whether an emergency physician is required at the scene based on the details provided in the emergency call. Ambulances are staffed with advanced paramedics (“Notfallsanitäter”) or emergency medical technicians (EMTs). Advanced paramedics complete a three-year training program and may independently perform selected interventions, including administration of life-saving drugs (e.g., adrenaline) in well-defined emergency situations, while most medications (opioids, cardiovascular agents) require physician involvement. Basic interventions such as oxygen or electrolyte administration may also be performed by other EMS personnel [16]. In addition to ambulances, physician-staffed ground or air units can be dispatched if required.

#### 2.2.4. Hospital Infrastructure

Hospitals in Germany are categorized as primary, secondary, or tertiary care centers according to size and range of services. Primary care hospitals offer basic inpatient services, mainly internal medicine and general surgery, and are often located in rural areas. Secondary care hospitals provide a broader range of specialties and serve as regional referral centers. Tertiary care hospitals, including university hospitals, offer highly specialized and complex services and are involved in teaching and research [17]. The study region is served by two primary care hospitals (Eichhof Hospital Lauterbach, Alsfeld Hospital), a secondary care facility (Schlüchtern Hospital), and tertiary care centers such as Fulda Clinic and the University Hospital of Giessen and Marburg.

### 2.3. Study Population

We included all EMS activations involving patients aged ≥65 years within the study period. Cases were classified as “inside NH” when EMS personnel selected the predefined NH category in the digital protocol; all other activations were categorized as “outside NH.” Duplicate records resulting from parallel documentation by ambulance and physician teams were excluded (*n* = 3786).

### 2.4. Data Collection

All prehospital patient care was documented electronically using tablets (NIDApad, Version 1.5.38.138, medDV GmbH, Fernwald, Germany). Reports were transmitted to a secure server and accessed as anonymized copies via VPN. Free-text medical history was not available for analysis due to data protection.

The dataset included demographic characteristics (age, sex), operational measures (response and on-scene times, lights and sirens use during dispatch and transport, physician dispatch), clinical severity (ABCDE-based assessment, feedback codes), and vital signs.

Feedback codes, transmitted to receiving hospitals before transport, summarized condition type and severity based on the extended ABCDE approach [18,19]. Severity levels were documented using predefined five-level scales integrated into the EMS tablet system (NIDApad), covering breathing, cardiovascular system, level of consciousness, neurological deficits, injuries, and pain. These scales represent a subjective impression of the patient’s condition and are not based on numerical thresholds. They range from very low (no impairment) to critical (life-threatening impairment). For example, in the breathing category, level 3 indicates moderate dyspnea, whereas level 5 denotes apnea (critical).

In contrast, the objectively measured vital signs were categorized in the Results section according to established literature-based thresholds: pulse <60 or >100 bpm, systolic blood pressure <100 or >140 mmHg, respiratory rate <12 or >20 per minute, and oxygen saturation <96% [18,19].

Response time was defined as the interval from call acceptance at the dispatch center to arrival on-scene; on-scene time was defined as interval from arrival on-scene to departure from the scene. Hospital admission urgency was categorized into four groups: 1 = immediate hospital intervention required, 2 = inpatient admission required, 3 = outpatient evaluation or diagnostic clarification and 0 = no admission required.

Missing values were transparently reported for each variable in the results section. The total number of cases reflects the complete available dataset within the defined period and setting.

### 2.5. Outcome Measures

Outcomes were differences between EMS responses in and outside NH regarding: travel and on-scene times, emergency physician dispatch, use of lights and sirens, ABCDE severity, feedback codes, hospital destination and admission urgency. Further outcome measures included symptom patterns, frequency of abnormal vital signs, and temporal variation in EMS activations.

### 2.6. Handling of Missing Data

Data completeness was reviewed for all variables prior to analysis. Demographic parameters such as age and sex were fully available. Automatically recorded vital signs (e.g., heart rate, blood pressure, oxygen saturation) showed very few missing values (<5%). Minor variations in totals for operational parameters (e.g., destination hospital, lights-and-sirens use, hospital admission urgency) reflected incomplete documentation in a small number of cases (<5%). Manually entered variables, particularly blood glucose, showed substantially higher missingness (~45–50%) due to the non-automated measurement process. Analyses were based on available cases only; missing data were not imputed.

### 2.7. Statistical Analysis

All eligible EMS responses for patients aged ≥65 years within the defined study period (July 2020–March 2025) were included; therefore, no a priori sample size calculation was performed. Before analysis, all variables were reviewed for completeness and plausibility.

Normality of continuous variables was assessed using the Shapiro–Wilk test. As most continuous variables were not normally distributed, comparisons between groups were performed using the Mann–Whitney U test. Continuous variables (e.g., age, travel time, and on-scene time) were summarized as median [IQR]. Effect sizes for these non-parametric comparisons were calculated as r = Z/√N and interpreted according to Cohen’s thresholds (small ≈ 0.10, medium ≈ 0.30, large ≈ 0.50) [20].

Categorical variables (e.g., sex, physician dispatches, and urgency of hospital admission) were compared using the Chi-square test or Fisher’s exact test when expected cell counts were <5. Corresponding effect sizes were expressed as Cramer’s V with 95% confidence intervals and interpreted according to Cohen’s conventions (small ≈ 0.10, medium ≈ 0.30, large ≈ 0.50) [20].

Analyses were based on available cases only; missing data were not imputed. All tests were two-sided, and *p* < 0.05 was considered statistically significant. Statistical analyses were performed using IBM SPSS Statistics for Mac, Version 30.0.0.0 (IBM Corp., Armonk, NY, USA) and Microsoft Excel 2013 (Microsoft Corp., Redmond, WA, USA).

## 3. Results

### 3.1. Number of Emergency Medical Responses

**Figure 1:**
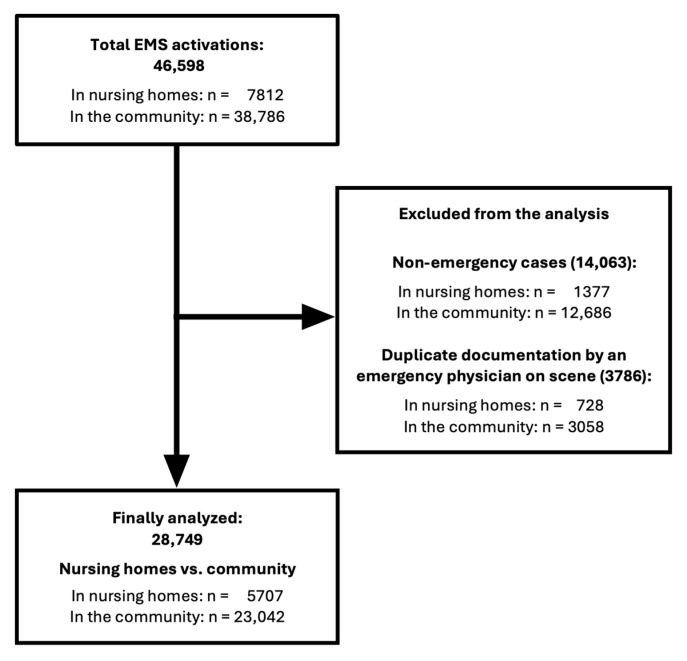
Flowchart of analysis process from total EMS responses involving patients aged ≥65 years (*n* = 46,598) to the final dataset. EMS = Emergency Medical Service.

A total of 46,598 EMS activations involving patients aged ≥65 years were recorded from July 2020 to March 2025, including 7812 in NHs and 38,786 in the community. Non-emergency transports (i.e., scheduled or physician-ordered patient transfers without an acute medical emergency) and duplicate records from emergency physician documentation were excluded, resulting in 28,749 analyzed emergency responses. Details of case selection are presented in Figure 1.

### 3.2. Statistics on Emergency Responses

**Figure 2:**
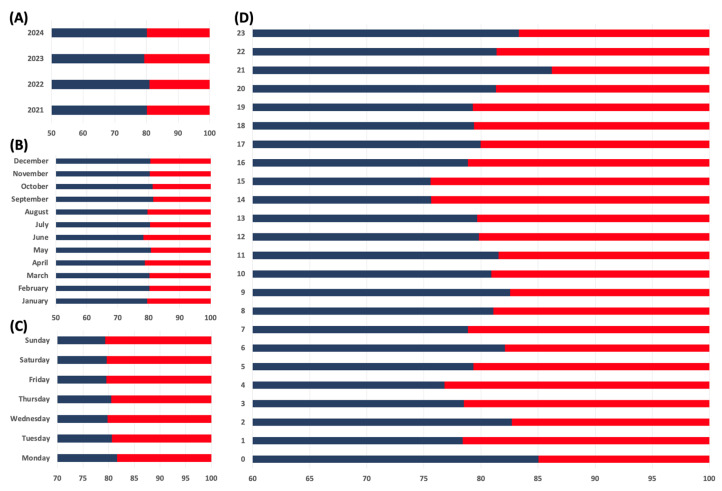
This figure illustrates the distribution of EMS responses to NHs (red bars) compared to those occurring outside NHs (blue bars). It shows the relative proportions of these responses over several dimensions. (**A**) Annual trends, (**B**) Monthly trends, (**C**) Weekly trends, (**D**) Hourly distribution. The figure highlights the proportion of EMS responses to NHs relative to the total number of responses, emphasizing the contribution of NH calls to the overall emergency workload.

Overall, approximately 20% of all EMS responses among patients aged ≥65 years (5707/28,749) occurred in NHs, while 80% (23,042/28,749) took place outside NHs. The distribution of EMS deployments by year, month, weekday, and daytime is shown in Figure 2. Hospital assignments were determined by feedback codes indicating clinical severity and by real-time hospital availability coordinated through the regional EMS dispatch center. Most NH patients were transported to primary care hospitals, with smaller proportions admitted to secondary or tertiary care facilities. A total of 16% were transferred to other regional institutions (Table 1). Similarly, the majority of patients outside NHs were also transported to primary care hospitals, while smaller proportions were admitted to secondary or tertiary care centers. In this group, 23% were transferred to other regional hospitals. In 710 cases, the receiving hospital was not recorded and therefore excluded from the hospital-level analysis. Detailed operational characteristics, including response times and transport times, emergency physician involvement, and hospital destinations, are summarized in Table 1 and Supplementary Table S1.

### 3.3. ABCDE Approach

The results for the ABCDE approach are reported according to existing categorization standards [18,19].

#### 3.3.1. A-Airway

No patient report indicated a case of complete airway obstruction.

#### 3.3.2. B-Breathing (Oxygen Saturation, Respiratory Rate, Auscultation Findings)

In 73% (4090/5618) of electronically documented records from EMS responses inside NHs, spontaneous breathing was described as unremarkable. A respiratory rate was documented in 94% (5347/5707) of cases. Among these, 81% (4306/5347) fell within the normal range of 12–20 breaths per minute. The median respiratory rate was 15 breaths per minute (IQR 14–18). Peripheral oxygen saturation (SpO_2_) was available in 93% (5322/5707) of records, with a median value of 94% (IQR 90–96).

In 79% (17,704/22,542) of electronically documented records from EMS responses outside NHs, spontaneous breathing was described as unremarkable. A respiratory rate was documented in 93% (21,445/23,042) of cases. Among these, 85% (18,230/21,445) fell within the normal range of 12–20 breaths per minute. The median respiratory rate was 15 breaths per minute (IQR 14–18). SpO_2_ was available in 94% (21,719/23,042) of records, with a median value of 95% (IQR 92–97). Additional respiratory findings are summarized in Supplementary Table S2.

#### 3.3.3. C-Circulation (Capillary Refill Time, Pulse Rate, Systolic Blood Pressure)

A pulse rate was documented in 95% (5401/5707) of NH cases and 95% (21,940/23,042) of community cases. The median pulse rate was 80 bpm (IQR 70–93) inside and 81 bpm (IQR 70–95) outside NHs, while the median systolic blood pressure was 131 mmHg (IQR 112–153) and 146 mmHg (IQR 126–168), respectively. Blood pressure values were documented in 89% (5075/5707) of NH and 92% (21,175/23,042) of community cases. Additional cardiovascular parameters, including heart rate categories and systolic blood pressure ranges, are summarized in Table 2.

#### 3.3.4. D-Disability (Neurological Status Assessed Using the AVPU Scale, Blood Glucose and Pain Score)

The majority of patients were documented as “alert” in both groups, though less frequently in NHs than in community settings (81% vs. 92%). Altered levels of consciousness (“only responsive to speech,” “responsive to pain,” or “unconscious”) were more common in NH residents. Further categories were documented in only a small proportion of cases (each <1%) and are therefore not described in detail. Detailed distributions are shown in Supplementary Table S3.

Blood glucose measurement was available in 52% (2952/5707) of NH cases and 55% (12,564/23,042) of community cases. The median blood glucose level was 7.8 mmol/L (6.3–10.0) in NHs and 7.3 mmol/L (6.1–9.1) outside NHs. Detailed distributions across blood glucose categories are presented in Supplementary Table S3. Pain was assessed using the Numeric Rating Scale (NRS), a standardized tool ranging from 0 (no pain) to 10 (worst possible pain) and was documented in most EMS responses. Moderate to severe pain (NRS ≥ 5) was recorded more frequently outside NHs than within. Pain scores were missing in 1% (56/5707) of NH cases and 5% (1146/23,042) of community cases. Detailed NRS distributions are presented in Supplementary Table S3.

#### 3.3.5. E-Exposure

An “unremarkable” skin condition was documented in 49% (2691/5509) of EMS responses in NHs and in 65% (14,246/22,060) outside NHs. “Reduced skin turgor” was noted in 39% (2120/5509) in NHs and in 24% (5190/22,060) outside. “Peripheral edema” was noted in 4% (243/5509) in NHs and 4% (773/22,060) outside, “cold sweat” was reported in 3% (140/5509) of cases in NHs and in 4% (859/22,060) outside. Less frequent values were observed in smaller proportions.

### 3.4. Feedback Code—Type and Severity of the Condition

Table 3 presents the five most frequent feedback codes among NH and community patients. Sex distribution is provided in Supplementary Table S4. Supplementary Table S5 summarizes the documented severity levels across vital sign domains, including breathing, cardiovascular status, level of consciousness, neurological deficits, injuries, and pain. These levels represent a subjective clinical impression of the patient’s condition, ranging from very low (no impairment) to critical (life-threatening impairment). Supplementary Table S6 displays the distribution of admission urgency categories.

## 4. Discussion

This retrospective analysis of EMS responses for patients aged ≥65 years in NHs and community settings in a rural district of central Germany (July 2020–March 2025) revealed distinct demographic, clinical, and operational differences between the two groups. Lights-and-sirens activations and emergency physician dispatches were significantly less frequent for NH calls. On-scene, NH patients were older and more frequently female. Feedback codes in NHs more frequently reflected low-to-moderate acuity, with respiratory and nonspecific symptoms predominating, whereas ACS and other cardiovascular emergencies were more common outside NHs. NH patients were more frequently classified as non-urgent or outpatient, while community cases required immediate hospital intervention significantly more often.

### 4.1. Demographic Differences

NH residents were older (median 85 vs. 80 years) and more often female. This aligns with national demographic data showing that the likelihood of institutional long-term care rises with age, and that women—due to greater life expectancy—are disproportionately represented in NHs, particularly from 75 years onwards [3,21].

### 4.2. Temporal Distribution of Deployments

Similarly to previous data, emergency calls were evenly distributed across weekdays, with a slight increase in community cases on Mondays, likely due to delayed recognition of weekend health issues [22,23]. Outpatient services are often unavailable until Monday, whereas trained NH staff and care protocols may help mitigate such backlogs. The circadian variation in EMS activations with an afternoon peak likely reflects increased overall activity during this period [24]. NH residents generally require assistance with daily activities, which increases interaction and mobility within the facility. Consequently, this may also elevate the risk of falls during active hours—a pattern consistent with the afternoon increase in fall-related EMS activations [25].

### 4.3. Operational Aspects

Shorter travel times to NHs may reflect EMS familiarity with these fixed and well-known facilities, whereas community calls can originate from any location within the district and therefore require greater reliance on local knowledge or navigation tools, which could contribute to longer travel distances and times [26,27]. However, when interpreting these data, it should be noted that time variables were recorded as whole minutes without seconds in the EMS documentation system, which may slightly overestimate apparent differences between groups. Although statistically significant, the calculated effect sizes indicate very small effects, suggesting that the median difference of one minute is likely of limited or no clinical relevance, except in highly time-critical scenarios.

Structural advantages—such as accessible documentation, clear responsibility hierarchies, barrier-free infrastructure—may also contribute to slightly shorter on-scene times, though the difference was probably not clinically relevant and is consistent with Sinclair et al. [28]. Studies from Sweden, the UK, and Saudi Arabia highlight limited access to patient information for timely prehospital decisions, underscoring the need for structured communication between NH staff, EMS, relatives, and primary care to reduce delays and support accurate decisions [29,30,31].

Emergency physicians were dispatched less often to NH incidents than to community cases, as most residents presented with stable vital signs, while acute cardiovascular emergencies were more frequent outside NHs. NH calls instead often involved conditions of mild to moderate severity, including falls, pneumonia, or nonspecific symptoms. Physician dispatch is known to be highly context-dependent and prone to both under- and overtriage [32,33].

The observed discrepancy between en route and transport use of lights and sirens, as shown in Supplementary Table S1, likely reflects differences in dispatch triage accuracy and subsequent on-scene reassessment. In community settings, lay callers may overestimate urgency, while trained nursing staff can usually provide more structured and clinically relevant information. Furthermore, pre-existing cognitive impairments in NH residents may mask acute changes, potentially leading to underestimation of urgency and less frequent lights-and-sirens responses. Such diagnostic uncertainty may represent a particular challenge for dispatchers, especially when assessing unspecific neurological symptoms [34]. In addition, the quality of caller information may strongly influence dispatch accuracy and safety-oriented overtriage is often observed to avoid missing critical cases, which may be reflected in our findings showing greater en route lights-and-sirens activation in community settings [35,36]. Once EMS professionals assess the situation on scene, many cases may be downgraded in urgency, which might explain the markedly lower proportion of transports conducted with lights and sirens in both groups. These patterns, consistent across sexes, may suggest that dispatch priority is likely influenced by caller information quality and safety-oriented overtriage aimed at avoiding missed critical cases.

Although prehospital mortality data were not included as a primary outcome, corresponding information could be derived from feedback codes in the EMS documentation. Among 28,749 EMS missions, deaths at the scene—including confirmed deaths, unsuccessful cardiopulmonary resuscitation (CPR), and CPR with return of spontaneous circulation (ROSC) but subsequent death—were documented in 171 community cases and 26 NH cases (<1% overall). As this distribution closely mirrors the relative number of EMS missions in both settings, on-scene mortality is unlikely to have meaningfully influenced the observed transport rates.

### 4.4. Clinical Presentations in Prehospital Care

Bronchitis/Pneumonia was the second most common feedback code among NH residents but was absent from the community’s top five. The higher prevalence in NHs may reflect aspiration risk, particularly in neurologically impaired patients, consistent with more frequent documentation of rales [37,38,39,40]. These findings underscore the potential value of aspiration prevention strategies in institutional care [41].

ACS was the second most common feedback code in community patients aged ≥65 years but absent from the NH top five, possibly reflecting a genuinely lower incidence in NHs or a high prevalence of atypical presentations. Multimorbidity, sensory changes, and cognitive impairment may further reduce diagnostic sensitivity [42,43]. Up to 44% of patients ≥75 years with ACS, including 40% with STEMI, do not report chest pain, underscoring the need for rapid prehospital ECG in NH residents—even without typical symptoms—to avoid missed infarctions and delays [42,44,45,46]. Specialized training for NH and EMS personnel, supported by checklists or decision-support tools, could help recognize atypical ACS in dementia patients and lower diagnostic thresholds [47]. Our binary “inside vs. outside NH” categorization did not capture other settings, such as primary care, where ACS may be detected before EMS involvement (e.g., point-of-care troponin), which may partly explain higher community proportions. Sex differences observed mirror studies from Norway, Spain, and the UK, showing higher myocardial infarction rates in men [48,49,50]. Accordingly, male community patients more often triggered EMS dispatch and lights-and-sirens transport, reflecting their greater likelihood of cardiovascular emergencies.

Neurological impairments were more frequent in NH residents, consistent with the higher prevalence of dementia, Parkinson’s disease, and prior strokes [51,52]. Such deficits can mask acute deterioration, complicate EMS assessments, and affect triage or resource allocation [34,53]. Accordingly, NH calls less often involve lights-and-sirens dispatch. These patterns suggest differences in perceived urgency but also raise concern that time-critical conditions may be under-recognized. Further research is needed to determine whether such operational factors delay identification and management of high-risk events.

Closed extremity injuries were significantly more frequent among NH residents, whereas facial/head injuries were not among the top five community feedback codes, suggesting distinct injury patterns. In NHs, fall risk factors include dizziness, walking aids, balance impairment, polypharmacy, psychotropic medication use, gait instability, sensory deficits, mood disorders, male sex, advanced age, and low T-POMA scores [54,55]. The predominance of women—more susceptible to osteoporosis-related fractures—likely amplifies risk [56,57]. In one cohort, annual fall incidence among NH residents was 37%, with 41% sustaining ≥2 falls per year; 28% of all falls caused injury and 6% led to fractures [55]. These findings emphasize the importance of fall-prevention strategies in institutional care [58].

### 4.5. Pain Management

Pain intensity (NRS ≥ 5) was reported in only 7% of NH vs. 14% of community deployments. This may reflect more routine analgesic medication use in NHs but also under-recognition in cognitively impaired residents, as NRS relies on self-report and non-verbal signs are often missed [22,59]. The European SHELTER study likewise showed persistent deficits in NH pain management [60]. These findings underscore the need for dementia-validated EMS pain tools to improve detection and treatment [22,60].

### 4.6. Hospital Transport Destination

NH residents were often transported to local general hospitals, partly due to the less acute conditions, whereas community patients with cardiovascular emergencies were more often referred to tertiary centers with cardiology services. Outcomes in time-critical conditions such as ACS or stroke depend on specialized care [61,62,63]. For frail NH residents, pragmatic considerations—such as proximity to relatives or the expected benefits of hospitalization—may favor nearby facilities [6,64]. However, unconscious ageism and undertriage may also reduce transfers to specialized centers despite clear indications [64,65,66], a pattern likely reinforced by NH residency.

### 4.7. Admission Urgency

In Germany, NHs provide long-term residential care for individuals with varying degrees of dependency [67]. Care is delivered around the clock by qualified nurses and assistants who provide personal and basic medical care, including medication administration, wound management, monitoring of vital signs, and coordination with general practitioners. Approximately 55% of nursing staff are registered nurses, and 45% are nursing assistants [68]. Unlike other healthcare systems, physicians are not based on site but visit regularly or on request. Medical interventions such as oxygen administration, intravenous fluids, or injections are legally permitted only under a physician’s explicit order [68]. In acute situations without such an order, nursing staff are not authorized to initiate treatment independently, and therefore EMS is contacted. Interprofessional collaboration in German long-term care is often hierarchical and documentation-based, with physicians making most clinical decisions [69]. Limited physician availability—particularly on weekends and in rural areas—further contributes to reliance on EMS [70,71]. These structural and legal conditions may partly explain the frequency of EMS calls for low-acuity conditions observed in our study.

This context helps to interpret the finding that a significantly higher proportion of NH residents were classified in category 3 (outpatient examination or diagnostic clarification). This aligns with Lienesch et al., showing that German out-of-hours medical services are underused despite their potential to prevent avoidable admissions [4]. The Australian EDDIE program likewise demonstrated that empowering NH staff to detect early deterioration reduced EMS calls and admissions by 19% [72]. In contrast, results from the US INTERACT II program were mixed, emphasizing the importance of training and implementation fidelity [73,74]. NH residents were also more often assigned to category 0 (no hospital admission), consistent with evidence that institutional care is associated with lower conveyance rates and shorter on-scene times, providing a safety net for safe discharge [28]. By contrast, community patients were more often classified as category 1 (immediate hospital intervention or physician contact), possibly reflecting earlier problem recognition by NH staff [72].

Taken together, these findings may provide a framework for comparison with regions that have similarly aged populations. While the proportion of adults aged ≥65 years in Germany slightly exceeds the European average and is even higher in our study region, Japan represents a “super-aged” society with 29% of its population aged ≥65 years [75,76]. Our findings indicate that EMS activations in NH were generally less acute and often involved conditions of low-to-moderate severity. These findings are consistent with observations from Japan, where nearly 69% of EMS calls from nursing facilities are classified as low-acuity, yet they dominate emergency responses among adults aged ≥65 years [77]. Similarly to our NH cohort, falls represent a major reason for EMS calls among older adults in Japan. In a recent Japanese analysis, approximately 11% of EMS calls involving adults aged ≥65 years were fall-related [78]. Given the ongoing demographic aging, this trend is expected to increase EMS demand and may similarly challenge the German system in the future. However, direct cross-national comparisons are limited because the organization of prehospital emergency services and long-term institutional care differs markedly between Germany and Japan [79,80].

### 4.8. Strengths and Limitations

A major strength of this study is its comprehensive inclusion of 46,598 EMS deployments for adults ≥65 years over multiple years in both NH and community settings within a defined rural district, enabling direct comparison of demographic, clinical, and operational characteristics. We deliberately selected a region with a high proportion of elderly residents, as such areas typically have a greater density of NHs. Moreover, predominantly rural regions often face limited healthcare infrastructure, which poses specific challenges for prehospital emergency care. Integration of dispatch data, clinical findings, and feedback codes provides a detailed, context-specific understanding of care needs in these populations.

However, several limitations should be considered when interpreting the results.

(1) The retrospective design relied on EMS documentation, which may vary across teams, and the database may contain incomplete or inaccurate data, potentially affecting reliability and external validity. Furthermore, time variables were available only as timestamps with hours and minutes (no seconds), which limits temporal precision and may exaggerate small statistical differences.
(2) The binary “NH vs. non-NH” classification did not capture other care environments (e.g., primary care practices, private homes) with potentially different patient characteristics and pathways to EMS activation.
(3) Due to data protection regulations, handwritten medical history records were not included, which may have led to omission of relevant clinical information.
(4) No hospital or emergency department follow-up data were available to confirm diagnoses, feedback codes, or admission appropriateness.
(5) Although the large dataset provides strong statistical power and a high level of representativeness, it also increases the likelihood of detecting statistically significant differences with limited clinical relevance. Therefore, the interpretation of findings focused on the clinical context rather than statistical significance alone.

Despite these limitations, the study provides important insights into EMS responses in NHs and community settings and provides a strong basis for further research to improve prehospital care for older adults.

## 5. Conclusions

NH calls were associated with fewer lights-and-sirens activations and less frequent emergency physician involvement. NH residents were older, more often female, and presented with low-to-moderate acuity conditions. Common diagnoses included closed extremity injuries and bronchitis/pneumonia, often accompanied by rales on auscultation. In contrast, ACS and other cardiovascular emergencies were more prevalent in the community, particularly among men, consistent with international evidence on sex-specific incidence. NH residents were more frequently classified as non-urgent or outpatient and transported to nearby hospitals, whereas community patients more often required immediate intervention and referral to tertiary centers. In summary, EMS responses for older adults differ in clinical presentations, operational patterns, and hospital pathways.

## Figures and Tables

**Table 1:** General characteristics, operational parameters, and hospital destinations of EMS responses inside and outside NHs. Categorical variables were compared using Chi-square tests, and continuous variables (reported as median [IQR]) were compared using the Mann–Whitney U test. All tests were two-sided; values with *p* < 0.05 were considered statistically significant. Effect sizes were expressed as Cramer’s V (categorical) and r (continuous). Lights and sirens en route V = 0.066, lights and sirens during transport V = 0.022, emergency physician dispatch V = 0.033, response time to scene r = 0.092, and on-scene time r = 0.027.

Variable	NH	Outside NH	*p*-Value
Sex			
Female	60% (3450/5705)	53% (12,223/23,036)	*p* < 0.001
Male	40% (2255/5705)	47% (10,813/23,036)	*p* < 0.001
Age (years)			
Median (IQR)	85 (80–89)	80 (73–86)	
Responding with lights and sirens en route			
Yes	30% (1716/5707)	38% (8762/23,042)	*p* < 0.001
No	70% (3991/5707)	62% (14,280/23,042)	*p* < 0.001
Transport to the hospital with lights and sirens			
Yes	13% (731/5707)	15% (3403/23,042)	*p* < 0.001
No	87% (4976/5707)	85% (19,639/23,042)	*p* < 0.001
Response time (travel time to scene) (minutes)			
Median (IQR)	6.0 (3.0–10.0)	7.0 (4.0–11.0)	*p* < 0.001
On-scene time (from arrival to departure) (minutes)			
Median (IQR)	27.0 (20.0–35.0)	28.0 (20.0–37.0)	*p* < 0.001
Emergency physicians			
Alarms to the scene	7% (392/5707)	10% (2327/23,042)	*p* < 0.001
Destination Hospital			
Primary care hospitals	66% (3675/5588)	58% (12,946/22,332)	*p* < 0.001
Secondary care hospitals	9% (493/5588)	6% (1362/22,332)	*p* < 0.001
Tertiary care hospitals	12% (647/5588)	16% (3637/22,332)	*p* < 0.001

IQR = Interquartile Range, NH = Nursing home.

**Table 2:** Circulatory parameters in EMS responses inside and outside NHs. Capillary refill time was considered prolonged when exceeding two seconds. Results are presented as absolute numbers and percentages for each parameter. Categorical variables were compared using Chi-square tests (two-sided, α = 0.05). Overall effect sizes (Cramer’s V) were 0.018 for pulse rate and 0.147 for systolic blood pressure, interpreted according to Cohen’s conventions (small ≈ 0.10, medium ≈ 0.30, large ≈ 0.50).

Variable	NH	Outside NH	*p*-Value
Circulation			
Prolonged capillary refill time	17% (964/5623)	12% (2768/22,389)	*p* < 0.001
Severe bleeding	1% (81/5597)	2% (360/22,308)	*p* = 0.372
Shock	3% (176/5609)	3% (557/22,355)	*p* = 0.007
Pulse rate			
Bradycardia (<60 bpm)	8% (436/5401)	8% (1684/21,940)	*p* = 0.328
Normal pulse rate (60–100 bpm)	76% (4097/5401)	75% (16,348/21,940)	*p* = 0.042
Tachycardia (>100 bpm)	16% (868/5401)	18% (3908/21,940)	*p* = 0.003
Blood pressure			
Blood pressure <100 mmHg	12% (587/5075)	6% (1249/21,175)	*p* < 0.001
Blood pressure 100–140 mmHg	51% (2574/5075)	38% (7998/21,175)	*p* < 0.001
Blood pressure >140 mmHg	38% (1914/5075)	56% (11,928/21,175)	*p* < 0.001

Bpm = Beats per minute, NH = Nursing home.

**Table 3:** Overview of the five most common feedback codes/diagnoses for emergency deployments within and outside NHs.

Five Most Common Feedback Codes/Diagnoses Inside and Outside NH				
	NH		Outside NH	
1.	Facial/Head Injury	9% (520/5707)	Nonspecific Symptoms	6% (1341/23,042)
2.	Bronchitis/Pneumonia	6% (354/5707)	ACS	5% (1249/23,042)
3.	Closed Extremity Injury	6% (338/5707)	Closed Extremity Injury	5% (1156/23,042)
4.	Nonspecific Symptoms	5% (301/5707)	Hypertension	5% (1092/23,042)
5.	Stroke/TIA/Bleeding < 6 h	4% (218/5707)	Stroke/TIA/Bleeding < 6 h	4% (990/23,042)

ACS = Acute coronary syndrome, NH = Nursing home, TIA = Transient ischemic attack.

## Data Availability

The data supporting the findings of this study are available from the authors; however, access is restricted as they were used with permission from the Department of Hazard Prevention and Emergency Service, District of Vogelsberg, Goldhelg 20, 36341, Lauterbach, Germany, for this study and are not publicly available. Data may be obtained from the authors upon reasonable request and with approval from both the Department of Hazard Prevention and the Medical Director of Emergency Medical Services.

## Supplementary Materials

The following supporting information can be downloaded at: https://www.mdpi.com/article/10.3390/healthcare13212806/s1, Table S1: Lights-and-Sirens Activation During EMS Response and Transport; Table S2: Airway and breathing assessments in EMS responses for adults aged ≥65 years inside and outside NHs; Table S3: Neurological status parameters in EMS responses inside and outside NHs; Table S4: Sex Distribution of the Five Most Common Diagnoses by Location; Table S5: Feedback Codes After Initial EMS Assessment; Table S6: Prehospital Assessment of Admission Urgency by Location

## Abbreviations

The following abbreviations are used in this manuscript:

ACS	Acute Coronary Syndrome
Bpm	Beats per minute
CPR	Cardiopulmonary Resuscitation
ECG	Electrocardiogram
EDDIE	Early Detection of Deterioration In Elderly Residents
EMS	Emergency Medical Services
EMTs	Emergency Medical Technicians
INTERACT	Interventions to Reduce Acute Care Transfers
IQR	Interquartile Range
NRS	Numerical Rating Scale
NH(s)	Nursing Home(s)
ROSC	Return of Spontaneous Circulation
STEMI	ST-Elevation Myocardial Infarction
STROBE	Strengthening the Reporting of Observational Studies in Epidemiology
TIA	Transient Ischemic Attack
T-POMA	Tinetti Performance-Oriented Mobility Assessment
UK	United Kingdom
US	United States (of America)
VPN	Virtual Private Network

## References

[1] Federal Statistical Office (Destatis) (2022). The Population Group of Older Adults Aged 65 Years and Over.

[2] Germany Atlas (2022). Population Growth Is Strongest in Southern and Northwestern Germany.

[3] Federal Statistical Office (Destatis) (2021). Press Release No. N 057 of 29 September 2021—Almost 6 Million Older People Live Alone.

[4] Lienesch P., Rothgang H., Gerhardus A., Wolf-Ostermann K., Hoffmann F., Czwikla J. (2025). Hospitalizations, emergency medical care utilization, and contacts with the regional on-call medical services among nursing home residents in Germany: A cross-sectional study in 44 nursing homes. BMC Health Serv. Res..

[5] Krammel M., Drahohs V., Hamp T., Lemoyne S., Grassmann D., Schreiber W., Sulzgruber P., Schnaubelt S. (2023). The Epidemiology of Pre-Hospital EMS Treatment of Geriatric Patients in the City of Vienna-An Overview. J. Clin. Med..

[6] Gaik C., Wulf H., Mann V., Humburg D., Vojnar B. (2025). Evaluation of emergency medical responses to nursing homes in a local area of Germany. BMC Emerg. Med..

[7] https://statistik.hessen.de/sites/statistik.hessen.de/files/2024-05/AI6_j23.pdf.

[8] Hessian State Historical Information System (LAGIS). https://www.lagis-hessen.de/en.

[9] Federal Statistical Office (Destatis) Development of the Population in Vogelsbergkreis from 1997 to 2023 [Graph]. https://de.statista.com/statistik/daten/studie/1171589/umfrage/entwicklung-der-gesamtbevoelkerung-im-vogelsbergkreis/.

[10] Federal Statistical Office (Destatis) Population Density in Germany from 1991 to 2023 (Inhabitants per km^2^) [Graph]. https://de.statista.com/statistik/daten/studie/440766/umfrage/bevoelkerungs-dichte-in-deutschland/.

[11] Statistical Offices of the Federal States and the Centre for Interdisciplinary Regional Research (ZEFIR). https://www.wegweiser-kommune.de/daten/demografische-entwicklung+vogelsbergkreis-lk+2016-2023+tabelle.

[12] Eurostat. https://ec.europa.eu/eurostat/statistics-explained/index.php?title=Population_structure_and_ageing.

[13] Population Structure Indicators at the National Level. https://ec.europa.eu/eurostat/databrowser/view/demo_pjanind/default/table?lang=en.

[14] Seblova J., Cimpoesu D., Khoury A., Revue E., Trenkler S. (2018). Prehospital emergency care systems in Europe—EuSEM prehospital section survey 2016. Eur. J. Emerg. Med..

[15] Lavery M.D., Aulakh A., Christian M.D. (2025). Benefits of targeted deployment of physician-led interprofessional pre-hospital teams on the care of critically Ill and injured patients: A systematic review and meta-analysis. Scand. J. Trauma Resusc. Emerg. Med..

[16] Bovenkerk S., Mueller A., Rossaint R., Czaplik M., Follmann A. (2025). Telemedicine via data glasses in CBRN protection suit-Evaluation of medical qualification and technical feasibility. PLoS ONE.

[17] Blumel M., Spranger A., Achstetter K., Maresso A., Busse R. (2020). Germany: Health System Review. Health Syst. Transit..

[18] Thim T., Krarup N.H., Grove E.L., Rohde C.V., Løfgren B. (2012). Initial assessment and treatment with the Airway, Breathing, Circulation, Disability, Exposure (ABCDE) approach. Int. J. Gen. Med..

[19] Bruinink L.J., Linders M., de Boode W.P., Fluit C.R.M.G., Hogeveen M. (2024). The ABCDE approach in critically ill patients: A scoping review of assessment tools, adherence and reported outcomes. Resusc. Plus.

[20] Chicco D., Sichenze A., Jurman G. (2025). A simple guide to the use of Student’s t-test, Mann-Whitney U test, Chi-squared test, and Kruskal-Wallis test in biostatistics. BioData Min..

[21] Federal Statistical Office (Destatis) (2025). Statistical Report—Life Tables—2022/2024. https://www.destatis.de/DE/Themen/Gesellschaft-Umwelt/Bevoelkerung/Sterbefaelle-Lebenserwartung/sterbetafel.html.

[22] Cantwell K., Morgans A., Smith K., Livingston M., Spelman T., Dietze P. (2015). Time of Day and Day of Week Trends in EMS Demand. Prehosp. Emerg. Care.

[23] Ong M.E., Ng F.S., Overton J., Yap S., Andresen D., Yong D.K., Lim S.H., Anantharaman V. (2009). Geographic-time distribution of ambulance calls in Singapore: Utility of geographic information system in ambulance deployment (CARE 3). Ann. Acad. Med. Singap..

[24] Helander M.E., Formica M.K., Bergen-Cico D.K. (2024). The Daily Patterns of Emergency Medical Events. J. Biol. Rhythms..

[25] Sheridan E., Wiseman J.M., Quatman C.E. (2024). Timing of emergency medical services activations for falls. Arch. Gerontol. Geriatr. Plus.

[26] van Mark A., Hallstein T., Holzgreve F., Groneberg D.A., Ohlendorf D. (2024). How do different navigation systems affect emergency response time? A prospective simulation study. BMJ Open.

[27] Gonzalez R.P., Cummings G.R., Mulekar M.S., Harlan S.M., Rodning C.B. (2009). Improving rural emergency medical service response time with global positioning system navigation. J. Trauma.

[28] Sinclair D.R., Charlton K., Stow D., Burrow E., Hanratty B. (2023). Care Home Residency and Its Association with Ambulance Service Workload. J. Am. Med. Dir. Assoc..

[29] Hedqvist A.T., Herrera M.J. (2025). Ambulance clinicians’ perspectives on interprofessional collaboration in prehospital emergency care for older patients with complex care needs: A mixed-methods study. BMC Geriatr..

[30] Zorab O., Robinson M., Endacott R. (2015). Are prehospital treatment or conveyance decisions affected by an ambulance crew’s ability to access a patient’s health information?. BMC Emerg. Med..

[31] Harthi N., Goodacre S., Sampson F.C., Binhotan M., Alotaibi A.S. (2025). Paramedics and emergency medical technicians’ perceptions of geriatric trauma care in Saudi Arabia. BMC Emerg. Med..

[32] Samdal M., Thorsen K., Graesli O., Sandberg M., Rehn M. (2021). Dispatch accuracy of physician-staffed emergency medical services in trauma care in south-east Norway: A retrospective observational study. Scand. J. Trauma Resusc. Emerg. Med..

[33] Strandqvist E., Olheden S., Backman A., Jornvall H., Backstrom D. (2023). Physician-staffed prehospital units: A retrospective follow-up from an urban area in Scandinavia. Int. J. Emerg. Med..

[34] Jamtli B., Svendsen E.J., Jorgensen T.M., Kramer-Johansen J., Hov M.R., Hardeland C. (2024). Factors affecting emergency medical dispatchers decision making in stroke calls—A qualitative study. BMC Emerg. Med..

[35] Dami F., Golay C., Pasquier M., Fuchs V., Carron P.N., Hugli O. (2015). Prehospital triage accuracy in a criteria based dispatch centre. BMC Emerg. Med..

[36] Garza A.G., Gratton M.C., Chen J.J., Carlson B. (2003). The accuracy of predicting cardiac arrest by emergency medical services dispatchers: The calling party effect. Acad. Emerg. Med..

[37] Manabe T., Teramoto S., Tamiya N., Okochi J., Hizawa N. (2015). Risk Factors for Aspiration Pneumonia in Older Adults. PLoS ONE.

[38] Langmore S.E., Skarupski K.A., Park P.S., Fries B.E. (2002). Predictors of aspiration pneumonia in nursing home residents. Dysphagia.

[39] Martinez-Pena G., Mimenza-Alvarado A.J., Aguilar-Navarro S.G. (2024). Dysphagia in older adults with mild cognitive impairment and dementia through fluoroscopic study with barium swallow in a memory clinic. Front. Neurol..

[40] Jarvinen H., Tolppanen A.M., Hartikainen S. (2023). Risk factors of pneumonia in persons with and without Alzheimer’s disease: A matched cohort study. BMC Geriatr..

[41] Chen S., Kent B., Cui Y. (2021). Interventions to prevent aspiration in older adults with dysphagia living in nursing homes: A scoping review. BMC Geriatr..

[42] Grosmaitre P., Le Vavasseur O., Yachouh E., Courtial Y., Jacob X., Meyran S., Lantelme P. (2013). Significance of atypical symptoms for the diagnosis and management of myocardial infarction in elderly patients admitted to emergency departments. Arch. Cardiovasc. Dis..

[43] Damluji A.A., Forman D.E., Wang T.Y., Chikwe J., Kunadian V., Rich M.W., Young B.A., Ii R.L.P., DeVon H.A., Alexander K.P. (2023). Management of Acute Coronary Syndrome in the Older Adult Population: A Scientific Statement From the American Heart Association. Circulation.

[44] Hajduk A.M., Saczynski J.S., Tsang S., Geda M.E., Dodson J.A., Ouellet G.M., Goldberg R.J., Chaudhry S.I. (2021). Presentation, Treatment, and Outcomes of Older Adults Hospitalized for Acute Myocardial Infarction According to Cognitive Status: The SILVER-AMI Study. Am. J. Med..

[45] Nanna M.G., Hajduk A.M., Krumholz H.M., Murphy T.E., Dreyer R.P., Alexander K.P., Geda M., Tsang S., Welty F.K., Safdar B. (2019). Sex-Based Differences in Presentation, Treatment, and Complications Among Older Adults Hospitalized for Acute Myocardial Infarction: The SILVER-AMI Study. Circ. Cardiovasc. Qual. Outcomes.

[46] Ouellet G.M., Geda M., Murphy T.E., Tsang S., Tinetti M.E., Chaudhry S.I. (2017). Prehospital Delay in Older Adults with Acute Myocardial Infarction: The ComprehenSIVe Evaluation of Risk Factors in Older Patients with Acute Myocardial Infarction Study. J. Am. Geriatr. Soc..

[47] Buswell M., Lumbard P., Prothero L., Lee C., Martin S., Fleming J., Goodman C. (2016). Unplanned, urgent and emergency care: What are the roles that EMS plays in providing for older people with dementia? An integrative review of policy, professional recommendations and evidence. Emerg. Med. J..

[48] Albrektsen G., Heuch I., Lochen M.L., Thelle D.S., Wilsgaard T., Njolstad I., Bønaa K.H. (2016). Lifelong Gender Gap in Risk of Incident Myocardial Infarction: The Tromso Study. JAMA Intern. Med..

[49] de Miguel-Yanes J.M., Jimenez-Garcia R., Hernandez-Barrera V., de Miguel-Diez J., Munoz-Rivas N., Mendez-Bailon M., Pérez-Farinós N., López-Herranz M., Lopez-de-Andres A. (2021). Sex Differences in the Incidence and Outcomes of Acute Myocardial Infarction in Spain, 2016–2018: A Matched-Pair Analysis. J. Clin. Med..

[50] Millett E.R.C., Peters S.A.E., Woodward M. (2018). Sex differences in risk factors for myocardial infarction: Cohort study of UK Biobank participants. BMJ.

[51] Buchanan R.J., Wang S., Huang C., Simpson P., Manyam B.V. (2002). Analyses of nursing home residents with Parkinson’s disease using the minimum data set. Park. Relat. Disord..

[52] Hoffmann F., Kaduszkiewicz H., Glaeske G., van den Bussche H., Koller D. (2014). Prevalence of dementia in nursing home and community-dwelling older adults in Germany. Aging Clin. Exp. Res..

[53] Eichinger M., Robb H.D.P., Scurr C., Tucker H., Heschl S., Peck G. (2021). Challenges in the PREHOSPITAL emergency management of geriatric trauma patients—A scoping review. Scand. J. Trauma Resusc. Emerg. Med..

[54] Shao L., Shi Y., Xie X.Y., Wang Z., Wang Z.A., Zhang J.E. (2023). Incidence and Risk Factors of Falls Among Older People in Nursing Homes: Systematic Review and Meta-Analysis. J. Am. Med. Dir. Assoc..

[55] Ferrara P., Monti C.C., Rozza D., Fornari C., Antonazzo I.C., Ferrara M.C., Bellelli G., Brandi M.L., Mantovani L.G., Mazzaglia G. (2025). Incidence and risk factors for falls among nursing home residents in Italy: A retrospective cohort study. Aging Clin. Exp. Res..

[56] Zarowitz B.J., Cheng L.I., Allen C., O’Shea T., Stolshek B. (2015). Osteoporosis prevalence and characteristics of treated and untreated nursing home residents with osteoporosis. J. Am. Med. Dir. Assoc..

[57] Zimmerman S.I., Girman C.J., Buie V.C., Chandler J., Hawkes W., Martin A., Holder L., Hebel J.R., Sloane P.D., Magaziner J. (1999). The prevalence of osteoporosis in nursing home residents. Osteoporos. Int..

[58] Albasha N., Ahern L., O’Mahony L., McCullagh R., Cornally N., McHugh S., Timmons S. (2023). Implementation strategies to support fall prevention interventions in long-term care facilities for older persons: A systematic review. BMC Geriatr..

[59] Bauer U., Pitzer S., Schreier M.M., Osterbrink J., Alzner R., Iglseder B. (2016). Pain treatment for nursing home residents differs according to cognitive state—A cross-sectional study. BMC Geriatr..

[60] Lukas A., Mayer B., Fialova D., Topinkova E., Gindin J., Onder G., Bernabei R., Nikolaus T., Denkinger M.D. (2013). Treatment of pain in European nursing homes: Results from the Services and Health for Elderly in Long TERm Care (SHELTER) study. J. Am. Med. Dir. Assoc..

[61] Cha J.J., Bae S., Park D.W., Park J.H., Hong S.J., Park S.M., Yu C.W., Rha S.W., Lim D.S., Suh S.Y. (2022). Clinical Outcomes in Patients With Delayed Hospitalization for Non-ST-Segment Elevation Myocardial Infarction. J. Am. Coll. Cardiol..

[62] De Luca G., Suryapranata H., Ottervanger J.P., Antman E.M. (2004). Time delay to treatment and mortality in primary angioplasty for acute myocardial infarction: Every minute of delay counts. Circulation.

[63] Bouckaert M., Lemmens R., Thijs V. (2009). Reducing prehospital delay in acute stroke. Nat. Rev. Neurol..

[64] Poncet C., Carron P.N., Darioli V., Zingg T., Ageron F.X. (2024). Prehospital undertriage of older injured patients in western Switzerland: An observational cross-sectional study. Scand. J. Trauma Resusc. Emerg. Med..

[65] Chang D.C., Bass R.R., Cornwell E.E., Mackenzie E.J. (2008). Undertriage of elderly trauma patients to state-designated trauma centers. Arch. Surg..

[66] Park J., Huh Y., Song S., Kim S., Yoo J., Jung K., Choi D. (2025). Undertriage of Severe Geriatric Trauma Patients: Who Are We Missing?. Yonsei Med. J..

[67] https://www.destatis.de/DE/Themen/Gesellschaft-Umwelt/Gesundheit/Pflege/_inhalt.html#_x2w2rwrhp.

[68] Heger D., Herr A., Lückemann M., Reichert A., Tycher L. (2025). Personnel shortages and the provision of long-term care: An empirical analysis of German nursing homes. Eur. J. Health Econ..

[69] Fassmer A.M., Pulst A., Spreckelsen O., Hoffmann F. (2020). Perspectives of general practitioners and nursing staff on acute hospital transfers of nursing home residents in Germany: Results of two cross-sectional studies. BMC Fam. Pract..

[70] Lemoyne S., Van Bastelaere J., Nackaerts S., Verdonck P., Monsieurs K., Schnaubelt S. (2024). Emergency physicians’ and nurses’ perception on the adequacy of emergency calls for nursing home residents: A non-interventional prospective study. Front. Med..

[71] May S., Jonas K., Fehler G.V., Zahn T., Heinze M., Muehlensiepen F. (2021). Challenges in current nursing home care in rural Germany and how they can be reduced by telehealth—An exploratory qualitative pre-post study. BMC Health Serv. Res..

[72] Carter H.E., Lee X.J., Dwyer T., O’Neill B., Jeffrey D., Doran C.M., Parkinson L., Osborne S.R., Reid-Searl K., Graves N. (2020). The effectiveness and cost effectiveness of a hospital avoidance program in a residential aged care facility: A prospective cohort study and modelled decision analysis. BMC Geriatr..

[73] Ouslander J.G., Bonner A., Herndon L., Shutes J. (2014). The Interventions to Reduce Acute Care Transfers (INTERACT) quality improvement program: An overview for medical directors and primary care clinicians in long term care. J. Am. Med. Dir. Assoc..

[74] Kane R.L., Huckfeldt P., Tappen R., Engstrom G., Rojido C., Newman D., Yang Z., Ouslander J.G. (2017). Effects of an Intervention to Reduce Hospitalizations From Nursing Homes: A Randomized Implementation Trial of the INTERACT Program. JAMA Intern. Med..

[75] Miyazaki R. (2023). Long-Term Care and the State-Family Nexus in Italy and Japan-The Welfare State, Care Policy and Family Caregivers. Int. J. Environ. Res. Public Health.

[76] Statistics Bureau of Japan. https://www.stat.go.jp/english/data/jinsui/2023np/index.html.

[77] Takayama Y., Hori A., Tanaka R., Ichikawa M. (2020). Ambulance use for low-acuity conditions by long-term care facilities for older adults. Eur. Geriatr. Med..

[78] Uemura S., Nakayama R., Koyama M., Taguchi Y., Bunya N., Sawamoto K., Ohnishi H., Narimatsu E. (2024). Prediction of the future number of fall-related emergency medical services calls in older individuals. Int. J. Emerg. Med..

[79] Yamada M., Arai H. (2020). Long-Term Care System in Japan. Ann. Geriatr. Med. Res..

[80] Bergmann J.M., Strobel A.M., Holle B., Palm R. (2020). Empirical development of a typology on residential long-term care units in Germany—Results of an exploratory multivariate data analysis. BMC Health Serv. Res..

