# Supplementary material for: Emergency Medical Service Responses for Older Adults: A Retrospective Observational Study Comparing Nursing Homes and the Community
Source: Healthcare (Basel). 2025 Nov 5;13(21):2806. doi: 10.3390/healthcare13212806 (PMC12609666; doi:10.3390/healthcare13212806)
Supplement: Supplementary file 1 [file healthcare-13-02806-s001.zip › healthcare-3887648-supplementary.pdf]

healthcare

MDPI

Supplementary Materials

Supplementary Table S1:

Lights and sirens		Male	Female	p-value
En route	NH	28 % (627/2,255)	32 % (1,087/3,450)	p=0.003
	Outside NH	40 % (4302/10,813)	37 % (4,457/12,223)	p<0.001
Transport	NH	13 % (292/2,255)	13 % (437/3,450)	p=0.786
	Outside NH	17 % (1801/10,813)	13 % (1,600/12,223)	p<0.001

Supplementary Table S1. Frequencies and percentages of lights-and-sirens use during EMS arrival and transport, stratified by sex and location. Frequencies were compared using Chi-square tests. All tests were two-sided; values with $p < 0.05$ were considered statistically significant. NH = Nursing home.

Supplementary Table S2:

Variable	NH	Outside NH	p-value
Airway			
Open	90 % (5,115/5707)	92 % (21,311/23,042)	p<0.001
Not assessable	0.16 % (9/5,707)	0.31 % (71/23,042)	p=0.073
Not examined	0.04 % (2/5,707)	0.15 % (34/23,042)	p=0.052
Other	10 % (581/5,707)	7 % (1,626/23,042)	p<0.001
Breathing			
(Unremarkable) Spontaneous breathing	72 % (4,090/5,708)	77 % (17,704/23,038)	p<0.001
Dyspnea	21 % (1,223/5,708)	17 % (3,885/23,038)	p<0.001
Rales	2 % (95/5,708)	1 % (187/23,038)	p<0.001
Other pathological breathing patterns	1 % (49/5,708)	1 % (140/23,038)	p=0.044

 https://doi.org/10.3390/healthcare13212806

Not documented	2 % (89/5,708)	2 % (500/23,038)	p<0.001

Supplementary Table S2: Airway and breathing assessments in EMS responses for adults aged ≥65 years inside and outside NHs. Group comparisons were performed using Chi-square tests. All tests were two-sided; values with p < 0.05 were considered statistically significant. NH = Nursing home.

Supplementary Table S3:

Variable	NH	Outside NH	p-value
Neurological status			
Alert	81 % (4,567/5,671)	92 % (20,916/22,684)	<0.001
Responsive to speech	12 % (670/5,671)	4 % (930/22,684)	<0.001
Responsive to pain	5 % (271/5,671)	1 % (272/22,684)	<0.001
Unconscious	1.4 % (80/5671)	1.5 % (342/22,684)	0.588
Blood glucose level			
Below 2.2 mmol/L	0.3 % (19/5,707)	0.4 % (85/23,042)	0.215
2.3 and 4.4 mmol/L	1 % (79/5,707)	1 % (293/23,042)	0.323
4.5 to 8.9 mmol/L	32 % (1,840/5,707)	38 % (8,815/23,042)	<0.001
> 8.9 mmol/L	18 % (1,014/5,707)	15 % (3,371/23,042)	<0.001
Pain (NRS)			
NRS between 0 and 4	93 % (5,281/5,707)	80 % (18,533/23,042)	<0.001
NRS between 5 and 10	7 % (370/5,707)	15 % (3,363/23,042)	<0.001

Supplementary Table S3. Neurological status parameters in EMS responses inside and outside NHs. Results are presented as absolute numbers and percentages for each parameter. Categorical variables were compared using Chi-square tests (two-sided, α = 0.05). Effect sizes (Cramer's V) were calculated for the overall distribution within each block: neurological status V = 0.160, blood glucose levels V = 0.054, and pain (NRS) V = 0.130. Effect sizes were interpreted according to Cohen's conventions (small ≈ 0.10, medium ≈ 0.30, large ≈ 0.50).

EMS = Emergency Medical Service, NRS = Numeric Rating Scale

Supplementary Table S4:

NH					
Variable	Male	Female	p-value	Cramer's V	95 % CI
Facial/Head Injury	39 % (202/520)	61 % (318/520)	p=0.604	0.219	0.160 - 0.276
Bronchitis/Pneumonia	46 % (164/354)	54 % (190/354)	p=0.014	0.060	0.001 - 0.133
Closed Extremity Injury	25 % (86/338)	75 % (252/338)	p<0.001	0.487	0.427 - 0.542
Nonspecific Symptoms	36 % (108/301)	64 % (193/301)	p=0.150	0.276	0.201 - 0.349
Stroke/TIA/Bleeding < 6 hours	31 % (67/218)	69 % (151/218)	p=0.005	0.378	0.295 - 0.456
Outside NH					
Variable	Male	Female	p-value	Cramer's V	95 % CI
Nonspecific Symptoms	45 % (605/1341)	55 % (736/1341)	p=0.162	0.095	0.057 - 0.132
ACS	51 % (633/1249)	49 % (616/1249)	p=0.008	0.001	0.001 - 0.039
Closed Extremity Injury	31 % (356/1156)	69 % (800/1156)	p<0.001	0.383	0.347 - 0.417
Hypertension	26 % (286/1092)	74 % (806/1092)	p<0.001	0.475	0.442 - 0.507
Stroke/TIA/Bleeding < 6 hours	49 % (484/990)	51 % (506/990)	p=0.236	0.001	0.001 - 0.044

Supplementary Table S4: Sex distribution for the five most common feedback codes/diagnoses for emergency deployments within and outside NHs. Group comparisons were conducted using Chi-square tests. All p-values are two-sided, and $p < 0.05$ was considered statistically significant. Effect sizes were calculated as Cramer's V with 95 % confidence intervals and interpreted according to Cohen's conventions (small ≈ 0.10, medium ≈ 0.30, large ≈ 0.50).

ACS = Acute coronary syndrome, NH = Nursing home, TIA = Transient ischemic attack

Supplementary Table S5:

Severity Level	Location of EMS Response		p-value
Breathing	NH (n=5,351)	Outside NH (n=21,092)	
Very low	64 % (3,433)	72 % (15,077)	p<0.001
Mild	23 % (1,249)	20 % (4,178)	p<0.001
Moderate	7 % (361)	4 % (844)	p<0.001
Severe	5 % (280)	4 % (783)	p<0.001
Critical	0 % (28)	1 % (210)	p=0.001
Cardiovascular System	NH (n=5,351)	Outside NH (n=21,112)	
Very low	52 % (2,756)	43 % (9,002)	p<0.001
Mild	38 % (2,037)	42 % (8,892)	p<0.001
Moderate	7 % (352)	10 % (2,104)	p<0.001
Severe	3 % (180)	4 % (903)	p=0.003
Critical	0 % (26)	1 % (211)	p<0.001
Level of Consciousness	NH (n=5,350)	Outside NH (n=21,098)	
Very low	74 % (3,961)	88 % (18,595)	p<0.001
Mild	22 % (1,166)	8 % (1,800)	p<0.001
Moderate	2 % (96)	1% (296)	p=0.040
Severe	1 % (74)	1 % (270)	p=0.597
Critical	1 % (53)	1 % (137)	p=0.011
Neurological Deficits	NH (n=5,344)	Outside NH (n=21,067)	
Very low	51 % (2,741)	78 % (16,343)	p<0.001
Mild	40 % (2,129)	12 % (2,601)	p<0.001
Moderate	6 % (293)	6 % (1,174)	p=0.824

Severe	3 % (169)	4 % (854)	p=0.003
Critical	0 % (12)	0 % (95)	p=0.027
Injuries	NH (n=5,351)	Outside NH (n=21,119)	
Very low	67 % (3,607)	76 % (16,092)	p<0.001
Mild	18 % (973)	12 % (2,477)	p<0.001
Moderate	11 % (604)	8 % (1,770)	p<0.001
Severe	3 % (166)	4 % (756)	p=0.097
Critical	0 % (1)	0 % (24)	p=0.077
Pain	NH (n=5,349)	Outside NH (n=21,104)	
Very low	60 % (3,193)	55 % (11,515)	p<0.001
Mild	28 % (1,470)	25 % (5,234)	p<0.001
Moderate	11 % (592)	15 % (3,191)	p<0.001
Severe	2 % (91)	5 % (1,077)	p<0.001
Critical	0 % (3)	0 % (87)	p<0.001

Supplementary Table S5: A feedback code was transmitted to the dispatch center for all 28,749 patients following the initial EMS assessment, summarizing the patient's condition and severity. The five-level scale reflects the subjective clinical impression of EMS personnel and ranges from very low (no impairment) to critical (life-threatening impairment). Data are presented as absolute numbers and percentages. Variations in totals reflect missing or incomplete documentation.
Group comparisons were conducted using Chi-square tests. All p-values are two-sided, and $p < 0.05$ was considered statistically significant. Effect sizes (Cramer's V) with 95 % confidence intervals were as follows: breathing 0.078 (95 % CI 0.066 - 0.090), cardiovascular system 0.078 (0.066 - 0.090), level of consciousness 0.171 (0.160 - 0.183), neurological deficits 0.290 (0.279 - 0.301), injuries 0.092 (0.080 - 0.104), and pain 0.088 (0.076 - 0.100), and were interpreted according to Cohen's conventions (small ≈ 0.10, medium ≈ 0.30, large ≈ 0.50).

NH = Nursing home, EMS = Emergency Medical Service

Supplementary Table S6:

Category	Urgency (for hospital admission)	NH	Outside NH	p-value
1	Immediate intervention in hospital, immediate contact with a physician	10 % (545/5,706)	13 % (2,886/23,037)	p<0.001
2	Inpatient admission likely, but no immediate action required	76 % (4,335/5,706)	75 % (17,233/23,037)	p<0.071
3	Outpatient treatment probably sufficient or diagnostic workup required, stay probably less than 24 hours	11 % (621/5,706)	7 % (1,664/23,037)	p<0.001
0	Non-urgent, no admission	4 % (205/5,706)	5 % (1,253/23,037)	p<0.001

Supplementary Table S6: Prehospital assessment of urgency for hospital admission among adults aged ≥65 years inside and outside NHs. The urgency level was determined by the EMS team on scene and coded on a scale from 0 to 3 (0 = non-urgent, no admission; 1 = immediate hospital intervention or physician contact; 2 = inpatient admission likely but no immediate action required; 3 = outpatient treatment or diagnostic workup sufficient, stay <24 h). Group differences were analyzed using Chi-square tests. All p-values are two-sided, and $p < 0.05$ was considered statistically significant. Effect size was calculated as Cramer's V = 0.070 (95 % CI 0.058 – 0.081) and interpreted according to Cohen's conventions (small ≈ 0.10, medium ≈ 0.30, large ≈ 0.50).

NH = Nursing home
